# Serious Rodent Bites to an 8-Month-Old Infant Due to Child Neglect

**DOI:** 10.7759/cureus.18493

**Published:** 2021-10-05

**Authors:** Konstantinos Skarentzos, Nikolaos Papadopulos, Savvas P Deftereos, Stavros Thomaidis, Katerina Kambouri

**Affiliations:** 1 Medicine, Democritus University of Thrace/General University Hospital of Alexandroupolis, Alexandroupolis, GRC; 2 Plastic Surgery, Democritus University of Thrace/General University Hospital of Alexandroupolis, Alexandroupolis, GRC; 3 Radiology, Democritus University of Thrace/University General Hospital of Alexandroupolis, Alexandroupolis, GRC; 4 Pediatrics, Democritus University of Thrace/University General Hospital of Alexandroupolis, Alexandroupolis, GRC; 5 Pediatric Surgery, Democritus University of Thrace/University General Hospital of Alexandroupolis, Alexandroupolis, GRC

**Keywords:** infant, child abuse and neglect, multiple bite injuries, plastic surgery, mice bites

## Abstract

Animal bites are among the top causes of preventable traumatic injuries. We describe the case of an 8-month-old female infant who was brought to the emergency department by her grandfather with serious rodent bites on her eyes, nose, right cheek, upper right extremity, and other smaller bites all over her body. This case is another proof of child neglect, or a possible infanticide attempt, as the motives of leaving the child in a hut without proper care, are not cleared up to this date. Rodent bites could be associated with Streptobacillus moniliformis infection and rat-bite fever. If the bites are left untreated for hours, the infant may suffer from hypovolemic shock due to bleeding, a possible fatal situation. The emergency surgical treatment of wounds is of vital importance.

## Introduction

Animal bites are among the top causes of preventable traumatic injuries. Children are most affected by animal bites [1]. However, accurate epidemiological information could not be obtained because not everyone who is bitten by an animal seeks medical care [2]. Rat bites are associated with poor living conditions in urban areas in the USA [3,4].

Another reason for rodent bites in the young population is child neglect. Child neglect results from several family factors such as gender bias, young age of the parents, poverty, etc. [5]. Herein, we describe a case of child neglect due to poverty and an incredibly young age of parents who could not recognize their infant’s needs and left her alone in a hut without heat and other household amenities for hours. Τhe infant was attacked by rodents which caused her multiple bite injuries.

## Case presentation

**Figure 1:**
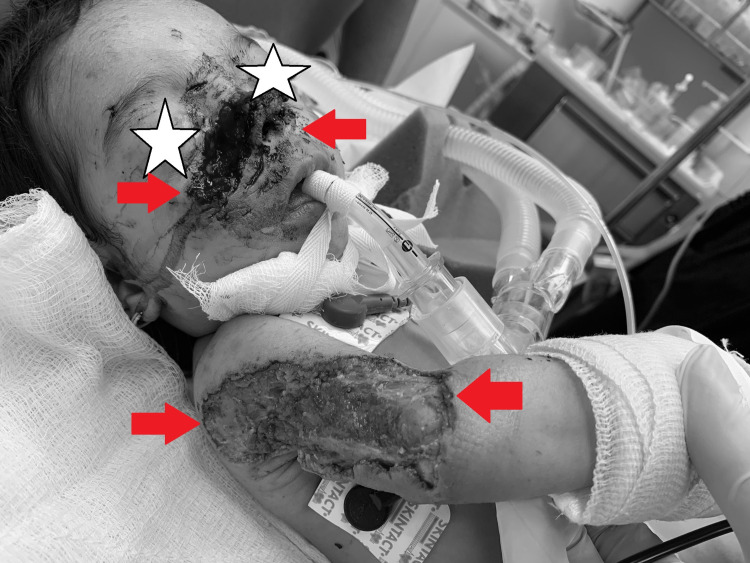
Infant before intervention

An 8-month-old female infant was brought to the pediatric emergency department with extensive injuries to her face, right arm, and other smaller injuries all over her body due to rat bites. Based on her medical history, she was brought to the hospital by her grandfather, who found her alone on the floor of the hut that her parents lived, without anyone near her. Grandfather pointed out that he saw her getting beaten by multiple mice before he rescued her. The child was actively bleeding from her right cheek and nose, which was injured, exposing her nasal orifices (Figure 1).

**Figure 2:**
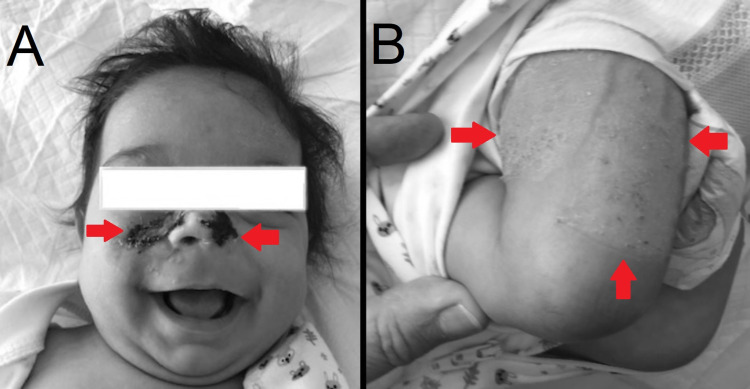
Infant after intervention

The child was hypothermic and hypovolemic. Fluid resuscitation, antitetanic toxoid, and ceftriaxone were initiated immediately, and a FAST (Focused Assessment with Sonography in Trauma) was performed to exclude intra-abdominal bleeding due to child abuse. After the initial stabilization of her vital signs, the child underwent surgical cleansing and partial suturing of the wound. Subsequently, she was admitted to the special baby care unit of the hospital. On the third postoperative day, her vital signs and temperature stabilized, she resumed eating, and all the cultures of her traumas were negative. Afterwards, it was deemed necessary to transfer the patient to a special plastic surgery unit to restore her wounds with grafts (Figure 2A, B).

## Discussion

Rodent bites are extremely rare in the Western world. In the United States from 2001 to 2015, there were 1,216 cases of rat-bite injuries, with an average annual rate of 0.27 per one million persons. Among them, only 21% were infants, children, or young adults (0-19 years old) [6]. It has been reported that the hands and head/face/neck regions are most affected by rat-bite injuries [7]. Our results are in agreement with this statement.

Nevertheless, poverty, unemployment, and poor housing are related to rodent bites [7]. This was confirmed in the present case. In addition, this infant was a victim of child neglect. A literature search was conducted to identify similar cases of child neglect. There was only one similar case in the last 10 years in the Western world [8], and one case series of two cases was identified in India [5]. The main cause of child neglect in India is the gender bias. In our case, the main cause of child neglect was the very young age of the parents and poverty. Rodent bite due to child neglect is an extremely rare incident in the western world, especially in Greece. To our knowledge, this is the first case of a child rat-bite injury in our hospital.

Rodent bites can transmit Streptobacillus moniliformis, a gram-negative bacterium that causes rat-bite fever. The clinical presentations included fever, polyarthritis, and rashes. Streptobacillus moniliformis culture requires a medium enriched with serum or ascites fluid. Therefore, if the requirements are not met, false-negative results may occur [9]. In our case, the cultures were negative, and no fever, polyarthritis, or rash was observed. Thus, hypovolemic shock may be related to bleeding and exposure to a cold environment.

This extremely rare case of rodent bite due to child neglect underlines the connection between child neglect and poverty. Another etiology that should be considered is the very young age of the parents. Teenagers may lack proper sexual education and appropriate family support, especially in families with low socioeconomic status. The possibility of infanticide attempts cannot be ruled out by the evidence, as the child was left unattended in the hut without proper care.

## Conclusions

Rat-bite injuries are rarely caused by child neglect. It could be associated with Streptobacillus moniliformis infection and rat-bite fever. If the bites are left untreated for hours, the infant may suffer from hypovolemic shock due to bleeding, a possible fatal situation. The emergency surgical treatment of wounds is of vital importance. Although the low socioeconomic status and the young age of the parents may have played a role, the reasons for leaving the infant in the hut are not clear up to this date. This case may reveal an infanticide attempt by parents. The motives for leaving an infant completely unattended in a hut, without heat, food, or water raises some concerns. 

## References

[1] Kannikeswaran N, Kamat D (2009). Mammalian bites. Clin Pediatr.

[2] Schalamon J, Ainoedhofer H, Singer G, Petnehazy T, Mayr J, Kiss K, Höllwarth ME (2006). Analysis of dog bites in children who are younger than 17 years. Pediatrics.

[3] Hirschhorn RB, Hodge RR (1999). Identification of risk factors in rat bite incidents involving humans. Pediatrics.

[4] Childs JE, McLafferty SL, Sadek R (1998). Epidemiology of rodent bites and prediction of rat infestation in New York City. Am J Epidemiol.

[5] Sethi SK, Saha A, Karela M, Dubey NK (2011). Infantile trauma due to a rat bite. J Emerg Trauma Shock.

[6] Kache PA, Person MK, Seeman SM, McQuiston JR, McCollum J, Traxler RM (2020). Rat-bite fever in the United States: an analysis using Multiple National Data Sources, 2001-2015. Open Forum Infect Dis.

[7] De Klerk P, Van Dijk M, Van As AB (2016). Treatment and outcome of unusual animal bite injuries in young children. S Afr Med J.

[8] Banerjee P, Ali Z, Fowler DR (2011). Rat bite fever, a fatal case of Streptobacillus moniliformis infection in a 14-month-old boy. J Forensic Sci.

[9] Graves MH, Janda JM (2001). Rat-bite fever (Streptobacillus moniliformis): a potential emerging disease. Int J Infect Dis.

